# Design of Plasmonic Photonic Crystal Fiber for Highly Sensitive Magnetic Field and Temperature Simultaneous Measurement

**DOI:** 10.3390/mi14091684

**Published:** 2023-08-29

**Authors:** Wenjun Zhou, Xi Qin, Ming Lv, Lifeng Qiu, Zhongjiang Chen, Fan Zhang

**Affiliations:** 1 Zhejiang Huayun Electric Power Engineering Design & Consultation Co., Ltd., Hangzhou 310014, China; xiqin1122023@163.com (X.Q.); MingLv123111@163.com (M.L.); qiulifeng2023@126.com (L.Q.); chenzhongjiang2023@126.com (Z.C.); 2 School of Automation, Hangzhou Dianzi University, Hangzhou 310000, China; zhangfan@hdu.edu.cn

**Keywords:** photonic crystal fiber, plasmonic, magnetic field, surface plasmon resonance, dual-parameter sensing

## Abstract

A high-sensitivity plasmonic photonic crystal fiber (PCF) sensor is designed and a metal thin film is embedded for achieving surface plasmon resonance (SPR), which can detect the magnetic field and temperature simultaneously. Within the plasmonic PCF sensor, the SPR sensing is accomplished by coating both the upper sensing channel (Ch1) and the lower sensing channel (Ch2) with gold film. In addition, the temperature-sensitive medium polydimethylsiloxane (PDMS) is chosen to fill in Ch1, allowing the sensor to respond to the temperature. The magnetic field-sensitive medium magnetic fluid (MF) is chosen to fill in Ch2, allowing this sensor to respond to the magnetic field. During these processes, this proposed SPR-PCF sensor can achieve dual-parameter sensing. The paper also investigates the electrical field characteristics, structural parameters and sensing performance using COMSOL. Finally, under the magnetic field range of 50–130 Oe, this sensor has magnetic field sensing sensitivities of 0 pm/Oe (Ch1) and 235 pm/Oe (Ch2). In addition, this paper also investigates the response of temperature. Under the temperature range of 20–40 °C, Ch1 and Ch2 have temperature sensitivities of −2000 pm/°C and 0 pm/°C, respectively. It is noteworthy that the two sensing channels respond to only a single physical parameter; this sensing performance is not common in dual-parameter sensing. Due to this sensing performance, it can be found that the magnetic field and temperature can be detected by this designed SPR-PCF sensor simultaneously without founding and calculating a sensing matrix. This sensing performance can solve the cross-sensitivity problem of magnetic field and temperature, thus reducing the measurement error. Since it can sense without a matrix, it further can solve the ill-conditioned matrix and nonlinear change in sensitivity problems in dual-parameter sensing. These excellent sensing capabilities are very important for carrying out multiparameter sensing in complicated environments.

## 1. Introduction

Humans have relied on the magnetic field as a key physical parameter to understand the world and explore undiscovered territory, and it has several applications in various geophysical domains. A magnetic field sensor is a part that transforms the magnetic field’s variation in strength into usable data, and they have become an indispensable part of many fields in society. With the rapid development of technology, current magnetic field sensors have the ability to detect different magnetic fields, which are highly used in many applications [1,2]. For instance, researchers can obtain pre-earthquake warnings by using magnetic detectors to capture weak, low-frequency magnetic field signals [3]. Utilizing magnetic field sensors and magnetic resonance imaging technologies, lesions may be seen, aiding medical professionals in the detection of diseases [4]. The use of magnetic exploration technology allows people to better understand the distribution of mineral deposits for precise mining [5]. In the field of marine safety, magnetic field sensors are also indispensable and important equipment [6,7].

Based on various technologies and principles, magnetic field sensors can currently be separated into electrical and optical fiber sensors. In electrical sensors, a Tesla meter has low sensitivity, and it cannot detect a weak magnetic field [8]. Inductive magnetometers are based on the Faraday effect; the sensitivity depends on the area and number of the coil, the permeability of the ferromagnetic core and the magnetic flux through the coil; and they do not apply to a magnetic field that is stationary. Using an electrical sensor made of a shield sector plate and a metal sheet, only the magnetic field can be measured at a single spot due to its weakness against electromagnetic noise [9,10]. Based on the magnetoresistive effect, magnetometers can detect the magnetic field, which includes anisotropic magnetoresistive, giant magnetoresistive, magnetic tunnel junction, extraordinary magnetoresistive, etc. [11,12,13]. Although these sensors are low-cost and easy to manufacture, they are affected by electromagnetic interference and have low sensitivities. Superconducting quantum interference devices can detect weak changes in external magnetic flux by measuring changes in the maximum superconducting current, which has a very high detection sensitivity [14]. However, it has a complex structure, huge size and expensive design and is relatively limited in practical applications. In conclusion, electrical magnetic field sensors are dependent on extremely demanding environments due to their small size, high sensitivity, strong resistance to electromagnetic interference, ease of use for remote sensing, integration and multiplexing. Over the past few decades, optical fiber magnetic field sensors have grown in popularity [15]. Optical fiber magnetic field sensors may be able to meet the needs of more applications compared with electrical magnetic field sensors, and they have also received a great deal of attention recently.

Nowadays, optical fiber magnetic field sensors include the Faraday effect [16,17], magnetostrictive effect [18,19] and magneto-refractive effect [20,21]. Sun et al. demonstrated an optical magnetic field sensor based on all-fiber, which consists of a 56-wt.% terbium-doped fiber Faraday rotator and a fiber polarized and has a sensitivity of 0.49 rad/T [16]. Shiyu et al. designed a tellurite glass multimode optical fiber for magneto-optical applications, and the sensor can measure 28.5 ± 0.5 rad/T·m [17]. From the results of these studies, the sensor based on the Faraday effect has a complex fabrication process and complicated signal demodulation. In magnetic field sensors based on the magnetostrictive effect, Zhan et al. designed a magnetic field sensor and experimentally demonstrated, in this sensor, that a Terfenol-D piece is bonded with the same type of two fiber Bragg gratings (FBGs) in a different direction with respect to the magnetic field. The magnetic field sensitivity of the suggested sensor is measured to be 8.77 pm/mT [18]. Peng et al. presented an optical fiber sensor with good repeatability and a high sensitivity of 9.83 pm/mT for magnetic field sensing combining an FBG with a magnetostrictive composite [19]. However, the high integration of magnetostrictive effect materials with optical fibers is a challenge. To achieve better magnetic field sensing, sensors based on the magneto-refractive effect have been focused on. These sensors usually use magnetic fluid (MF) and are designed with a special structure that makes the optical field of the core mode interact with MF. The refractive index tunability of MF allows these sensors to respond to the magnetic field. In general, this type of sensor has the advantage of high integration, simplicity, affordability, versatility, etc. For instance, Zhao et al. designed a hollow-core optical fiber, the cavity of which is filled with MF, and this sensor has a magnetic field sensitivity of 33 pm/Oe [20]. Luo et al. designed a structure with a magnetic field sensitivity of 65.9 pm/Oe and a lateral-offset fusion splicing between the main structure and the lead-out single-mode fiber. A very short segment of multimode fiber is sandwiched between two pieces of single-mode fiber in this construction [21]. Despite having some advantages in detecting the magnetic field, these optical fiber sensors are not sensitive enough. Additionally, magnetic nanoparticles in MF have peculiar kinetic features that make them sensitive to both temperature and magnetic field, so pondering the cross-sensitivity effect for these dual-parameter sensors is crucial [22]. Measurement of the magnetic field alone can lead to large errors in detection. 

In particular, the excited surface plasmon polariton (SPP) mode brought on by the surface plasmon resonance (SPR) effect generates superior sensing effects, making the SPR sensor promising [23,24]. Since SPR is a peculiar optical phenomenon [25], the SPR effect can establish the relationship between the absorbed light and the analyte to be measured. Compared to interferometric optical fiber sensors, SPR optical fiber sensors do not need to accumulate phase (increase the length) to increase sensitivity. The sensitivity mainly depends on the characteristics of the SPP mode in the analyte, so the type of SPR optical fiber sensors is highly expandable, and it can maintain both miniaturization and high sensitivity [26]. In addition, photonic crystal fiber (PCF) is a new type of optical fiber formed by introducing a periodic arrangement of air holes into silicon oxide artificially [27]. Different arrangements of air holes will provide different optical characteristics of PCF. The combination of PCF and SPR is expected to achieve excellent sensing performance [28]. 

In this paper, a high-sensitivity plasmonic PCF sensor is designed, and a metal thin film is embedded for achieving SPR, which can detect the magnetic field and temperature simultaneously. Within the plasmonic PCF sensor, the upper sensing channel (Ch1) and the lower sensing channel (Ch2) are both coated with gold film in order to achieve SPR sensing. In addition, Ch1 and Ch2 of the sensor are filled with polydimethylsiloxane (PDMS) and MF, respectively, which can make the proposed sensor respond to the magnetic field and temperature. By using COMSOL, this paper discusses and analyzes the mode characteristics, structural parameters, and sensing capabilities of the magnetic field and temperature. The numerical simulation data indicate that for this designed sensor, the y-polarized core mode (y-pol core mode) can mainly contribute to the excitation of SPR, and the characteristics of SPR can be controlled by adjusting the structural parameters of this sensor. Finally, the optimized sensor has magnetic field sensing sensitivities of 0 pm/Oe (Ch1) and 235 pm/Oe (Ch2). The related measuring range of the magnetic field is 50–130 Oe. In addition, this paper also investigates the response of temperature. Under the temperature range of 20–40 °C, Ch1 and Ch2 have temperature sensitivities of −2000 pm/°C and 0 pm/°C, respectively. Two sensing channels can respond to the magnetic field and temperature separately; this sensing performance is not common in dual-parameter sensing. This paper also explains why this phenomenon occurs through the optical properties of MF and the dispersion curve of the SPP mode. Due to this sensing performance, it can be found that this sensing performance not only solves the cross-sensitivity problem but also further solves the ill-conditioned matrix and nonlinear change in sensitivity problems in dual-parameter sensing with a sensing matrix [29]. 

## 2. Model and Theory

**Figure 1:**
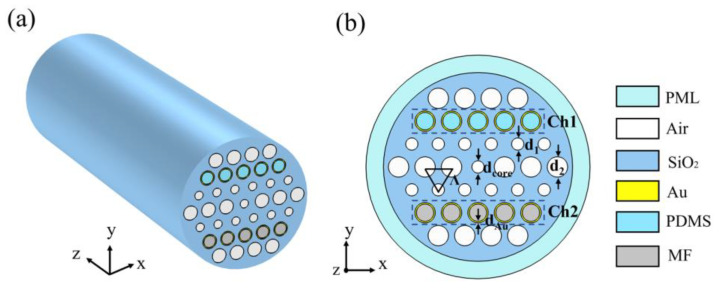
(**a**) Schematic three-dimensional diagram of the proposed sensor. (**b**) Schematic diagram of the proposed sensor in its cross-section.

A three-dimensional schematic diagram of the SPR-PCF sensor is shown in Figure 1a, and the stake-and-draw method can be used to construct the PCF [30]. The high-pressure chemical vapor deposition method or the technique of dynamic chemical liquid phase deposition enables specific air holes of the PCF to be deposited with nanomaterials [31,32,33,34]. Moreover, the selective filling process provides a method for filling any air hole with MF and PDMS [35,36]. By completing these processes, the designed SPR-PCF sensor can be manufactured. In this paper, the proposed sensor is simulated by using COMSOL Multiphysics; Figure 1b shows the cross-section schematic diagram of the sensor. The sensor has a lot of air holes, which reduces the effective refractive index of cladding and allows the core to confine the optical field. In order to reduce unneeded electromagnetic reflection in simulation, a perfectly matched layer (PML) is used as the absorbing border condition [37,38]. The structural parameters *d_Au_*, *d*_1_, *d*_2_, *d_core_* and *Λ* represent, accordingly, the gold film thickness; the diameter of the air holes in the second layer; the diameter of the air holes in the first, third and fourth layers; the diameter of the central air hole; and the pitch of the air hole. Among them, *d_1_* and *d_2_* are determined to be 1.2 μm and 2 μm, respectively, and the rest of them are analyzed and discussed subsequently. To react to the temperature, Ch1 is filled with PDMS after being coated with gold film. To react to the magnetic field, Ch2 is coated with gold film and then filled with MF. Additionally, the refractive indices of two media can alter depending on the temperature and magnetic field. Finally, temperature and magnetic field sensing can be achieved by observing the variation in the resonant wavelength of the SPR peak.

In this paper, the refractive index of air holes is set to 1. And the Sellmeier equation is used to express the refractive index of SiO_2_ [39]:

(1) $$n^{2}\left(\lambda \right)=1+\frac{B_{1}\lambda^{2}}{\lambda^{2}-C_{1}}+\frac{B_{2}\lambda^{2}}{\lambda^{2}-C_{2}}+\frac{B_{3}\lambda^{2}}{\lambda^{2}-C_{3}}$$

where λ is wavelength and the constants $B_{1}$, $B_{2}$, $B_{3}$, $C_{1}$, $C_{2}$ and $C_{3}$ in the Sellmeier equation are 0.6961663, 0.4079426, 0.8974794, 0.0684043 μm, 0.1162414 μm and 9.896161 μm, respectively. 

The Drude–Lorentz model is used to calculate the relative dielectric constant of the gold layer [40]:

(2) $$\varepsilon_{m}=\varepsilon_{\infty }-\frac{\omega_{D}^{2}}{\omega (\omega +j\gamma_{D})}+\frac{\Delta \varepsilon \cdot \Omega_{L}^{2}}{\left(\omega^{2}-\Omega_{L}^{2}\right)+j\Gamma_{L}\omega }$$

where $\varepsilon_{\infty }$ is the dielectric constant at high frequency, $\omega_{D}$ is the plasma frequency of the electron, ω is the angular frequency of the incident light, $\gamma_{D}$ is the damping frequency, Δε is the spectral width of the Lorentz oscillators, $\Omega_{L}$ is the oscillator strength and $\Gamma_{L}$ represents the weighting factor.

**Figure 2:**
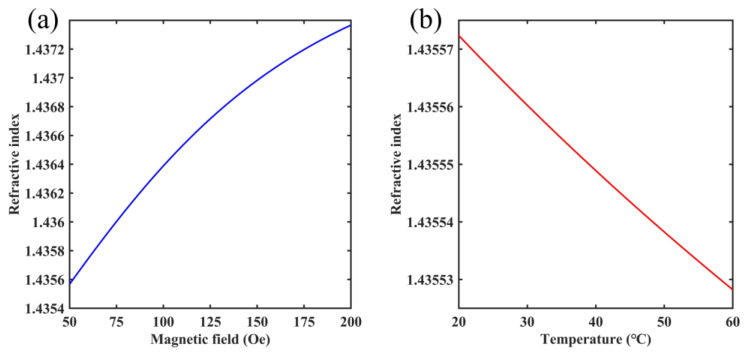
(**a**) The change in refractive index of MF with the magnetic field. (**b**) The change in refractive index of MF with the temperature.

MF is a stable colloidal solution, which is composed of magnetic nanoparticles with magnetic properties uniformly dispersing in a based solution under the action of surfactants. Due to kinetic factors of magnetic nanoparticles, MF is affected by both the magnetic field and temperature. This property of MF is described by the following Langevin function [41]:

(3) $$n_{mf}=(n_{s}-n_{0})[\coth(\alpha \frac{H-H_{c,n}}{T})-\frac{T}{\alpha (H-H_{c,n})}]+n_{0}$$

where $n_{s}$ is the saturation value of the refractive index, $n_{0}$ is the initial refractive index of MF, H is the external ambient magnetic field and $H_{c,n}$ is the threshold range for the magnetic field. When H is larger than $H_{c,n}$, this paper can obtain the refractive index of MF. In addition, α is the MF fitting coefficient, and T is the external ambient temperature. MF used in this paper is the water-based Fe_3_O_4_, and the parameters for MF are set to T = 24.3 °C, α = 5, $H_{c,n}$ = 30 Oe, $n_{0}$ = 1.4352 and $n_{s}$ = 1.4385 [42]. As shown in Figure 2, this paper shows the refractive index variation characteristics of MF with the magnetic field and temperature changes. It can be found that the refractive index of MF increases with the magnetic field and decreases with the temperature. Therefore, this type of sensor needs to take into account the effect of temperature during the testing of the magnetic field. In addition, the change in the refractive index due to the magnetic field and temperature is reversed, and the temperature crosstalk is expected to be directly suppressed by utilizing the nonlinear change in sensitivity for the SPR sensor.

In order to perform temperature sensing, PDMS, as a polymer material with a high thermo-optical coefficient and ease of processing, can be used in combination with PCF. Equation (4) can be used to express the relationship between its refractive index and temperature [43]:

(4) $$n_{\mathrm{PDMS}}=-4.5\times 10^{-4}T+1.4176$$

In this equation, *T* is the external ambient temperature (°C).

The loss characteristic is important data in the simulation for the SPR-PCF sensor. When the SPR effect occurs, the energy of the core mode is coupled to the SPP mode so that the SPR peak can be observed in the spectrum. The loss spectrum can be calculated using the following equation, and its characteristics can be used to assess how well the proposed sensor performs when it comes to sensing [44]:

(5) $$\alpha_{loss}=8.686\times \frac{2\pi }{\lambda }Im\left(n_{eff}\right)\times 10^{7}(\mathrm{dB}/\mathrm{cm})$$

where λ is wavelength and $Im\left(n_{eff}\right)$ is the imaginary part of the effective refractive index of the core mode. According to this equation, the loss spectrum is related to the length, but the length here is the length of the metal film. In practice, the length of the metal film can be shortened to improve the signal-to-noise ratio, and this operation does not affect the sensitivity basically.

The refractive index of MF or PDMS changes under the action of the external magnetic field or temperature, this effect further causes a change in the effective refractive index of the SPP mode on the surface of the gold film, the resonant wavelength of the SPR peak can shift. Therefore, the sensitivity of this sensor is obtained by computing a shift of the resonant wavelength when the refractive index of the analyte changes, which can be calculated by using the following equation [45]:

(6) $$S_{\lambda }=\frac{\Delta \lambda_{peak}}{\Delta n_{a}}(\mathrm{nm}/\mathrm{RIU})$$

where $\Delta \lambda_{peak}$ is the amount of shift of resonant wavelength and $\Delta n_{a}$ is the amount of change in the refractive index of the analyte. In this paper, the analyte includes MF and PDMS.

## 3. Analysis Mode Characteristics

**Figure 3:**
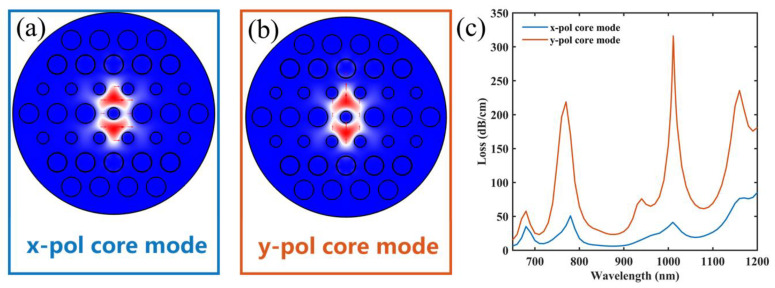
The electrical field distributions of (**a**) x polarization and (**b**) y polarization. (**c**) The loss properties on the spectrum of two core modes.

Using COMSOL, this paper can study the mode characteristics of the sensor. Figure 3a,b show the optical field distribution of the x-pol core mode and y-pol core mode. The x-polarized core mode (x-pol core mode) and y-pol core mode in the core have different effective refractive indices because the lack of rotational symmetry in the construction of this sensor causes the birefringence effect [46]. In addition, according to the SPP mode characteristics [47], different modes with different electrical field distributions have different degrees of excitation on the SPR effect. The coupling properties of the two core modes and the SPP mode can be seen in the loss spectra. Therefore, the loss spectra are first discussed in this paper. In Figure 3c, compared to the loss spectrum of the x-polarized core mode, the loss spectrum of the y-polarized core mode has a larger loss. This is mainly due to the fact that the excitation of the SPR effect depends on the electrical field in the direction of vibration perpendicular to the gold film. For this SPR-PCF sensor, the y-pol core mode contains a larger number of electrical field components that are perpendicular to the gold film compared to the x-pol core mode. Based on the previous discussion, the more energy of the y-pol core mode that is coupled to the SPP mode, the higher the sensitivity of the SPR peak excited by the y-pol core mode [48]. This phenomenon supports the choice of the y-pol core mode for the subsequent study in this paper. 

**Figure 4:**
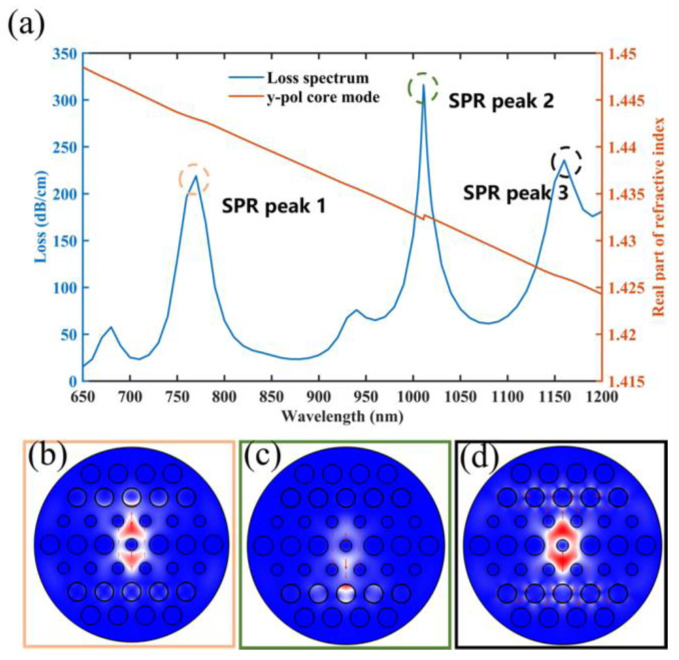
(**a**) Both the effective refractive index and the loss spectrum of the y-pol core mode. (**b**–**d**) The electrical field distributions of the y-pol core mode at 770 nm, 1011 nm and 1160 nm.

The loss spectrum and effective refractive indices of the y-pol core mode are shown in Figure 4a. First, since PDMS and MF filled in Ch1 and Ch2 have different refractive indices, the different SPP modes have different effective refractive indices in two sensing channels, thus producing two SPR peaks at 770 nm and 1011 nm, which are called SPR peak 1 and SPR peak 2, respectively. In addition, the circular-shaped sensing channels support the generation of a higher-order SPP mode. This is the reason for the third SPR peak at 1160 nm, which is called SPR peak 3. In addition, to illustrate the sensing performance of three SPR peaks, the electrical field distribution of the y-pol core mode at 770 nm, 1011 nm and 1160 nm is shown in Figure 4b–d. Most of the energy of the y-pol core mode is coupled with the SPP mode in Ch1 at 770 nm, which can generate SPR peak 1, which can respond to temperature. The majority of the coupling energy is transferred to the SPP mode in Ch2 at 1011 nm, and further considering the magneto-optical properties of MF, it becomes evident that the magnetic field and temperature can be measured by SPR peak 2. In Ch1 and Ch2, the coupling energy transferred to the SPP mode is basically equal at 1160 nm. However, in Figure 4d, it can be seen that most of the electrical field of the SPP mode is distributed in SiO_2_, and it is difficult for MF or PDMS to modulate the effective refractive index of this SPP mode, resulting in essentially no shift in SPR peak 3. Through the analysis, this paper examines the features of these two SPR peaks for future investigation, and the sensitivity of SPR peak 3 is not calculated. However, SPR peak 2 may be affected by SPR peak 3 during the optimization of the structural parameters of this sensor, so SPR peak 3 still has to be included in the subsequent study.

## 4. Analysis of Structural Parameters

The structural properties of the proposed sensor are improved in this part to enhance sensing capabilities. For this designed dual-parameter sensor, two main points are considered in this paper, which include the wavelength gap and overall loss of two SPR peaks. When the wavelength gap is increased, the dynamic detection range of the sensor can be improved [49,50]. In addition, the overall loss can reflect the coupling strength between the y-pol core mode and the SPP mode This means that by coupling more energy from the y-pol core mode to the SPP mode, the sensitivity of the sensor can be improved.

### 4.1. The Gold Film Thickness

**Figure 5:**
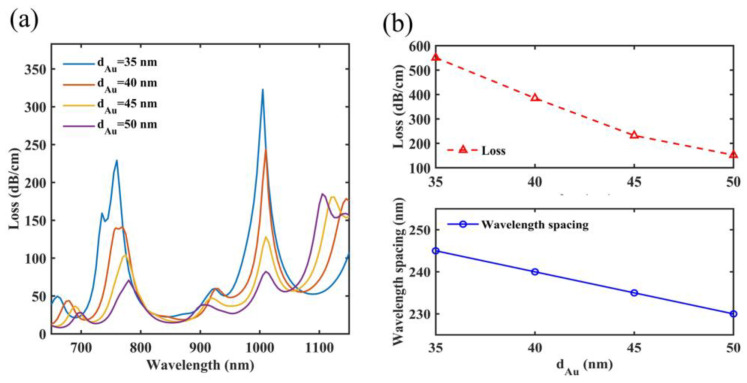
When *d_Au_* ranges from 35 nm to 50 nm, (**a**) the loss characteristics on the spectra of *d_Au_* and (**b**) two SPR peaks have different overall losses and wavelength gaps.

First, it is evaluated how the gold film thickness *d_Au_* affects sensing performance. Figure 5a shows that the loss characteristics have differences in the spectra when *d_Au_* ranges from 35 nm to 50 nm. The resonant wavelength of SPR peak 1 shifts from 760 nm to 780 nm, and the resonant wavelength of SPR peak 2 shifts from 1005 nm to 1010 nm. The main reason is that *d_Au_* affects the effective refractive index of the SPP mode, and then it can make the SPR peak move. In addition, because the dispersion curve of the SPP mode is nonlinear, its characteristics are different at different wavelengths, resulting in a different move of resonant wavelengths of two SPR peaks. Furthermore, because of the high intrinsic loss of the metal, the y-pol core mode loses its energy as it propagates through the gold film [51]. As *d_Au_* increases, the thicker gold layer needs to be penetrated by the y-pol core mode to allow coupling with the SPP mode [52]. In this process, the energy of the y-pol core mode is lost to a greater extent, so it can be seen that on the spectra, the overall loss of the discussed two SPR peaks is reduced with the increase in *d_Au_*. In Figure 5b, this paper calculates the overall losses and wavelength gaps, and the sensor has overall losses of 551 dB/cm, 385 dB/cm, 232 dB/cm and 152 dB/cm and wavelength gaps of 245 nm, 240 nm, 235 nm and 230 nm. In this paper, the increase in *d_Au_* will decline the performance of the sensor. However, considering the feasibility and tolerance of the process, *d_Au_* is chosen as 40 nm.

### 4.2. The Diameter of the Central Air Hole

**Figure 6:**
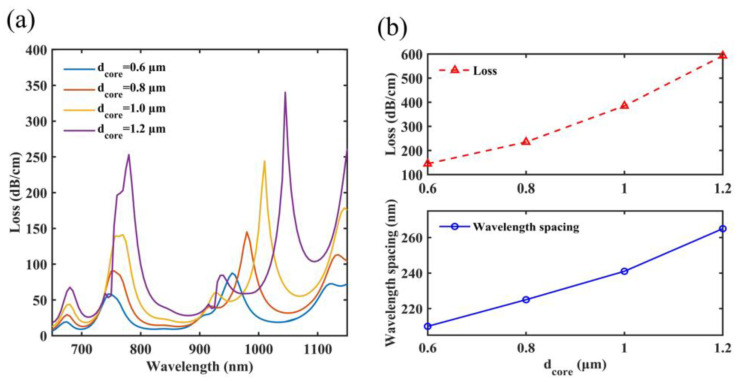
When *d_core_* ranges from 0.6 μm to 1.2 μm, (**a**) the loss characteristics on the spectra of *d_core_* (**b**) two SPR peaks have different overall losses and wavelength gaps.

Then, the impact of the diameter of the central air hole *d_core_* on sensing capacities has also been studied, which can be shown in Figure 6a,b. The refractive index of the central air hole is lower than the refractive index of the cladding; as a result, the effective refractive index of the y-pol core mode decreases as *d_core_* increases. The SPP mode is essentially unaffected because Ch1 and Ch2 are located far from the center. Finally, it is found that the resonant wavelength of SPR peak 1 shifts from 745 nm to 780 nm and the resonant wavelength of SPR peak 2 changes from 955 nm to 1045 nm on the spectra when *d_core_* is between 0.6 μm and 1.2 μm. In addition, as *d_core_* increases, the electrical field of the fiber core is closer to the gold film, which makes it easier to couple the energy of the y-pol core mode to the SPP mode [53]. Based on the trend, it seems that as *d_core_* increases, the better the performance of the sensor. But when *d_core_* exceeds 1.2 μm, the loss of SPR peak 3 is larger than that of SPR peak 2, and the two peaks are also close to each other, making it difficult to distinguish between SPR peak 2 and SPR peak 3. Therefore, the maximum value of *d_core_* for the study is 1.2 μm. It can be found in Figure 6b that the overall loss and wavelength gaps increase as *d_core_* increases. The sensor has overall losses of 145 dB/cm, 235 dB/cm, 385 dB/cm and 593 dB/cm. The wavelength gaps are, accordingly, 210 nm, 225 nm, 241 nm and 265 nm. After analyzing, *d_core_* can be set at 1.2 μm.

### 4.3. The Pitch of the Air Hole

**Figure 7:**
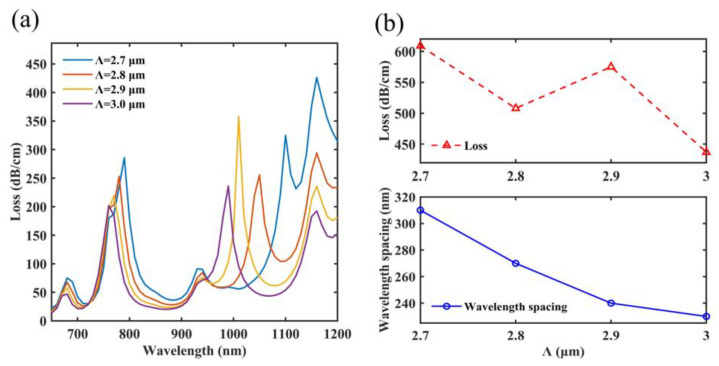
When *Λ* ranges from 2.7 μm to 3 μm, (**a**) the loss characteristics on the spectra of *Λ*, (**b**) two SPR peaks have a different overall loss and wavelength gap.

This paper also analyzes and optimizes the pitch of the air hole *Λ*. In Figure 7a, the resonant wavelength of SPR peak 1 shifts from 790 nm to 760 nm, whereas the resonant wavelength of SPR peak 2 shifts from 1100 nm to 990 nm. When *Λ* ranges from 2.7 μm to 3 μm, the percentage of SiO_2_ at Ch1, Ch2 and the fiber core will increase. The effective refractive indices of the SPP mode and the y-pol core mode increase as a result of this. The y-pol core mode is more impacted than the SPP mode because it has a greater electrical field in SiO_2_. This is the reason why the resonant wavelengths blue shift [54]. In addition, *Λ* can affect the ability of the designed PCF to confine the energy of the y-pol core mode. Therefore, changing *Λ* causes the overall loss to change. In Figure 7b, when *Λ* ranges from 2.7 μm to 3 μm, the overall losses are, accordingly, 609 dB/cm, 508 dB/cm, 575 dB/cm and 437 dB/cm. In addition, the sensor has wavelength gaps of 310 nm, 270 nm, 240 nm and 230 nm. The sensing performance is better when *Λ* is 2.7 μm, but SPR peak 2 is too close to SPR peak 3 and the loss of SPR peak 3 is larger than the loss of SPR peak 2. When the designed sensor performs magnetic field sensing, SPR peak 2 will be covered by SPR peak 3. Therefore, *Λ* is chosen as 2.9 μm. At this point, the sensor can maintain a high sensitivity and a large dynamic monitoring range.

## 5. Measurement of Magnetic Field and Temperature

**Figure 8:**
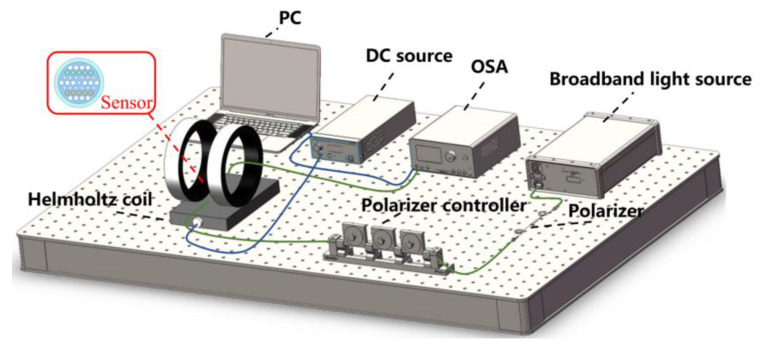
Schematic diagram of magnetic field test system.

Finally, after discussing these structural parameters, this paper tests the response characteristics of the magnetic field and temperature. To further illustrate the feasibility, a schematic diagram of the magnetic field test system is designed. Figure 8 illustrates the test procedure for the magnetic field using the designed sensor, and this system includes a broadband light source, a polarizer, a polarizer controller, a Helmholtz coil, an OSA, a DC source and a PC. The sensor is first placed on the Helmholtz coil. A broadband light source can generate the incident light at the wavelength required to excite the SPR effect. A polarizer and a polarizer controller can modulate the polarization characteristic of the incident light to excite the SPR effect. This Helmholtz coil can be controlled by the DC power to generate the different magnetic field, resulting in the resonant wavelength of SPR peak shifting [55]. Finally, the OSA and PC are used to receive and analyze the loss spectra and calibrate the sensitivity of the designed sensor.

**Figure 9:**
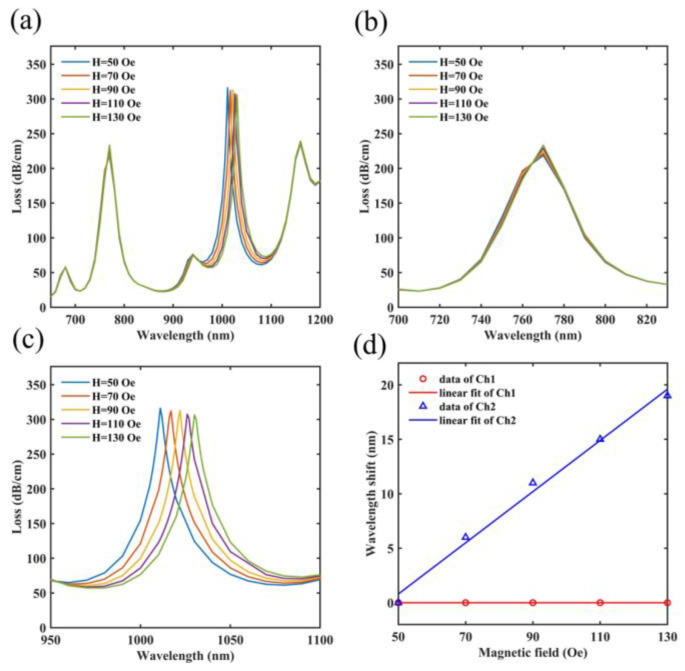
(**a**) Influence of the magnetic field ranging from 50 Oe to 130 Oe on the loss spectra of the optimized sensor. (**b**) An expanded perspective of SPR peak 1 on the spectra. (**c**) An expanded perspective of SPR peak 2 on the spectra. (**d**) Under varying magnetic fields, the fitted results of the resonant wavelengths of two SPR peaks.

Figure 9 depicts the simulation data of magnetic field sensing performance over the magnetic field range of 50–130 Oe. The unique magneto-optical properties of MF cause its refractive index to increase with the increasing magnetic field. This phenomenon makes the effective refractive index of the SPP mode in Ch2 increase. The resonant wavelength of SPR peak 2 red shifts as a result. In addition, the refractive index of PDMS is affected by the magnetic field. The resonant wavelength of the SPR peak 1 does not move. The reason for a small variation in the loss value is mainly from a little different degree of coupling between the y-pol core mode and the SPP mode in Ch2 under different magnetic fields. In Figure 9b,c, this paper also gives expanded perspectives of these two SPR peaks in the different magnetic fields on the spectra, based on which it can be found that the resonant wavelength of SPR peak 1 does not shift and the resonant wavelength of SPR peak 2 shifts from 1011 nm to 1030 nm. In addition, the resonant wavelength of SPR peak 2 remains a linear variation. The reason for this linear variation is that the refractive index of MF changes linearly in this magnetic field range, which makes the magnetic field sensitivity of the designed sensor linear. In Figure 9d, this paper calculates the magnetic field sensitivities of Ch1 and Ch2. The magnetic field sensitivity of Ch1 is 0 pm/Oe. In addition, Ch2 has the fitted result of $\Delta \lambda_{2}=0.235\times \Delta H-10.95$, so Ch2 can be used for detecting the magnetic field with a sensitivity of 235 pm/Oe. 

**Figure 10:**
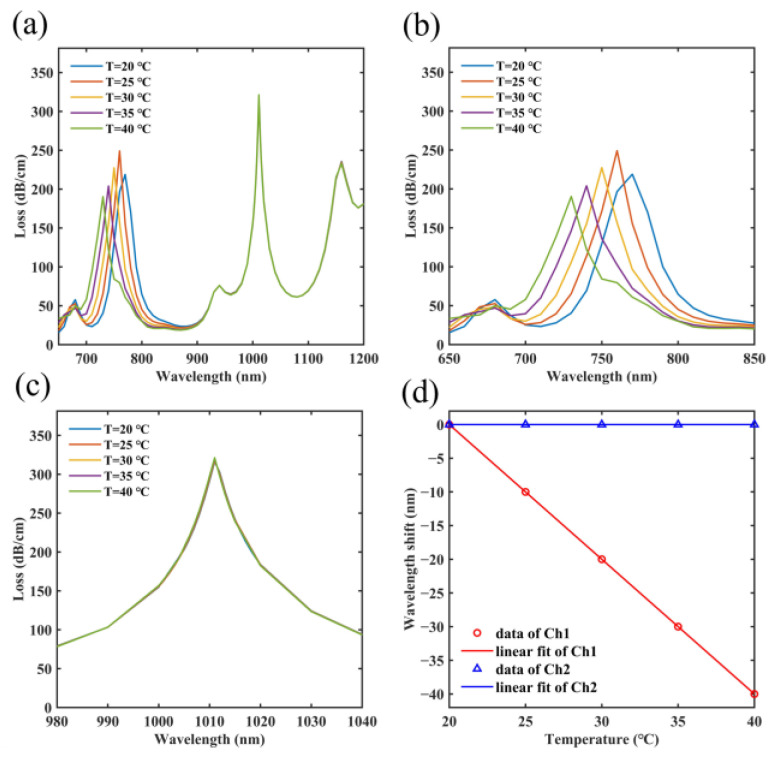
(**a**) Influence of the temperature ranging from 20 °C to 40 °C on the loss spectra of the optimized sensor. (**b**) Expanded perspective of SPR peak 1 on the spectra. (**c**) Expanded perspective of SPR peak 2 on the spectra. (**d**) Under varying temperatures, the fitted results of the resonant wavelengths of two SPR peaks.

Next, the optimized sensor is evaluated for its sensing performance within the temperature range of 20 °C to 40 °C, and the temperature sensing data are calculated in Figure 10. In Figure 10a, it can be found that the resonant wavelength of SPR peak 1 blue shifts significantly, whereas the resonant wavelength of SPR peak 2 remains unchanged. By referring to Equations (3) and (4), it can be observed that the temperature rise leads to a decrease in the refractive indices of MF and PDMS. PDMS is a high thermo-optical effect material, and the change in its refractive index is very sensitive to the temperature, resulting in a significant change in SPR peak 1 [50]. Compared to PDMS, the response of MF to temperature is relatively weak. Additionally, the SPP mode exhibits a nonlinear dispersion curve, leading to varying sensitivity of SPR peak 2 at different wavelengths. Therefore, the change in SPR peak 2 is not significant. Figure 10b,c displays expanded perspectives of these SPR peaks, revealing that the resonant wavelength of SPR peak 1 shifts from 770 nm to 730 nm with temperature variations. Furthermore, the resonant wavelength of SPR peak 2 does not move and stays at 1011 nm. Additionally, a linear fitted result is conducted to analyze the temperature sensitivities of both channels. Figure 10d illustrates that Ch1 produces a result of $\Delta \lambda_{1}=-2\times \Delta T+40$, indicating a temperature sensitivity of −2000 pm/°C for Ch1. The resonant wavelength of SPR peak 2 barely moves, resulting in a temperature sensitivity of 0 pm/°C for Ch2.

**Figure 11:**
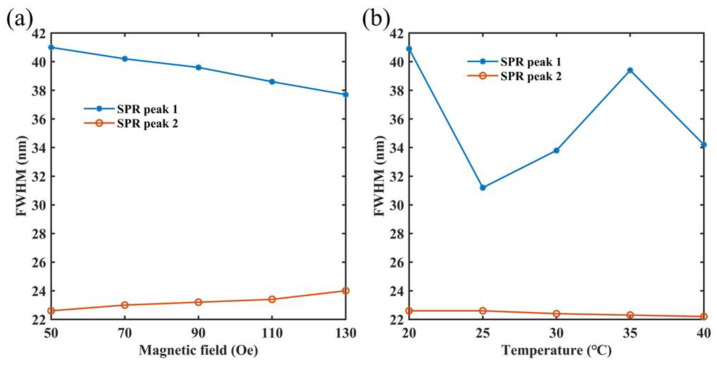
(**a**) The impact on FWHM of two SPR peaks when the magnetic field is adjusted between 50 Oe and 130 Oe. (**b**) The impact on FWHM of two SPR peaks when the temperature is adjusted between 20 °C to 40 °C.

Because of its excellent sensing performance, this paper also calculates the full width at half-maximum (FWHM) of two SPR peaks at different magnetic fields and temperatures, and Figure 11 shows the results. When this sensor detects the magnetic field, the FWHM of SPR peak 1 is 41 nm, 40.2 nm, 39.6 nm, 38.6 nm and 37.7 nm, and the FWHM of SPR peak 2 is 22.6 nm, 23 nm, 23.2 nm, 23.4 nm and 24 nm. It can be observed that FWHM does not change significantly. This is mainly due to the smaller spacing of magnetic field variations, resulting in smaller modulation effects on the SPR peaks and, therefore, smaller variations in the SPR peaks under different magnetic fields. In contrast, when the sensor detects the temperature, the FWHM of SPR peak 1 can change significantly. This is due to the temperature having a significant impact on the refractive index of PDMS, which in turn has a significant impact on the SPR peak 1. SPR peak 2 is resistant to temperature interference, so its FWHM remains largely unchanged. And the FWHM of SPR peak 1 is 40.9 nm, 31.2 nm, 33.8 nm, 39.4 nm and 34.2 nm, and the FWHM of SPR peak 2 is 22.6 nm, 22.6 nm, 22.4 nm, 22.3 nm and 22.2 nm. In general, the designed sensor retains a small FWHM, which is important for improving the detection limit of the sensor.

**Figure 12:**
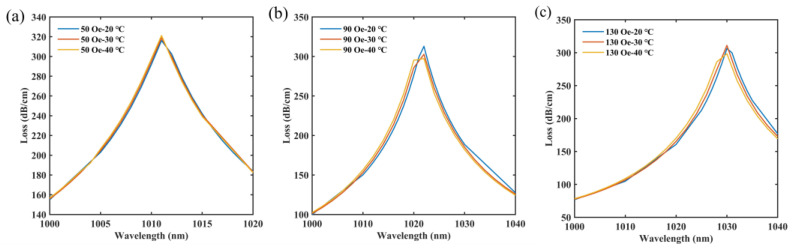
The effect of temperature variation or different temperature ranges on the magnetic sensing accuracy. (**a**) Under the magnetic field of 50 Oe and the temperature range from 20 to 40 °C; (**b**) Under the magnetic field of 90 Oe and the temperature range from 20 to 40 °C; (**c**) Under the magnetic field of 130 Oe and the temperature range from 20 to 40 °C.

In practice, the refractive index of MF will be also affected by the temperature. It is necessary to discuss the effect of temperature variation or different temperature ranges on magnetic sensing accuracy [56]. Since SPR peak 1 responds only to temperature, this paper investigates the immunity of SPR peak 2. As shown in Figure 12, under the different magnetic fields and temperatures, it can be found that SPR peak 2 is largely unaffected by the temperature. This further illustrates that SPR peaks generated by the designed sensor all respond to only a single physical parameter, thus allowing dual-parameter sensing of the magnetic field and temperature without demodulating a sensing matrix.

Through examination of the capacity of this proposed sensor for magnetic field and temperature detection, it becomes evident that the two SPR peaks respond to these two physical parameters correspondingly. This sensing performance not only resolves the issue of cross-sensitivity but also effectively addresses the problems of ill-conditioned matrix and nonlinear sensitivity changes in dual-parameter sensing. Finally, the paper investigates reported dual-parameter SPR-PCF sensors for the magnetic field and temperature in Table 1 [57,58,59,60,61,62,63]. Compared with them, the proposed sensor has excellent dual-parameter sensing sensitivity. In order to maintain a linear variation in sensitivity, the magnetic field and temperature intervals studied in this paper are not large, but it does not mean that the designed sensor has less sensitivity than the reported sensors of this table in larger test intervals. In addition, it is worth mentioning that the proposed sensor can respond to only a single physical parameter separately, so dual-parameter sensing of the magnetic field and temperature can be achieved without a sensing matrix. Within the reported sensors, they still require solving a sensing matrix to realize dual-parameter sensing, which can have many problems, including nonlinearly varying sensitivities that cannot be solved for with a matrix, ill-conditioned matrix and so on. Although some reported sensors can simultaneously excite SPR and achieve dual-parameter sensing through two polarized modes, they are complex to operate in practice (repeatedly adjusting the polarizer controller) [64,65]. In contrast, the designed sensor has the potential to broaden the scope of applications for optical sensors due to its high sensitivity and unique sensing capabilities.

## 6. Conclusions

In conclusion, a dual-parameter SPR-PCF sensor has been proposed, which can achieve highly sensitive magnetic field and temperature dual-parameter sensing and solve the temperature cross-sensitivity problem. The proposed sensor is simulated by using COMSOL, and its mode characteristics, structural parameters and sensing performance of the magnetic field and temperature are analyzed. The numerical simulation results show that the SPR effect excited by the y-pol core mode has better sensing performance than the x-pol core mode. In addition, the unique field distribution characteristics of SPR peak 1 and SPR peak 2 make the sensor respond to both the magnetic field and temperature. Then, the structural parameters *d_Au_*, *d_core_* and *Λ* of the sensor are optimized to achieve a better dual-parameter sensing performance. Finally, the optimized sensor has magnetic field sensing sensitivities of 0 pm/Oe (Ch1) and 235 pm/Oe (Ch2). The corresponding measurement range of magnetic field strength is 50–130 Oe. In addition, the response of temperature is also investigated. When the temperature is in the range of 20–40 °C, Ch1 and Ch2 have temperature sensitivities of −2000 pm/°C and 0 pm/°C, respectively. The SPR-PCF sensor achieves excellent sensing performance, which not only solves the cross-sensitivity problem but also further solves the ill-conditioned matrix and nonlinear change in sensitivity problems in dual-parameter sensing. Owing to its unique structure and excellent sensing characteristics, the present sensor has the potential to perform multiparameter sensing in complex environments.

## Figures and Tables

**Table 1:** Comparison of properties of dual-parameter SPR-PCF sensor reported.

Reference	Magnetic Field Sensitivity	Temperature Sensitivity	Response Range
[57]	60 pm/Oe	−4090 pm/°C	0–350 Oe/20–50 °C
[58]	77.9 pm/Oe	−1151 pm/°C	25–200 Oe/20–70 °C
[59]	108 pm/Oe	−226.9 pm/°C	0–600 Oe/0–80 °C
[60]	142.74 pm/Oe	−229 pm/°C	0–350 Oe/25–55 °C
[61]	164.06 pm/Oe	−5001.31 pm/°C	20~550 Oe/5–55 °C
[62]	44 pm/Oe	−370 pm/°C	0~500 Oe/20–50 °C
[63]	65 pm/Oe	−2360 pm/°C	50–130 Oe/17.5–27.5 °C
This work	235 pm/Oe	−2000 pm/°C	50–130 Oe/20–40 °C

## Data Availability

Publicly available datasets were analyzed in this study. These data can be found here: [https://www.lumerical.com/ (accessed on 1 January 2020)].
